# Multiphoton Fluorescence Microscopy with GRIN Objective Aberration Correction by Low Order Adaptive Optics

**DOI:** 10.1371/journal.pone.0022321

**Published:** 2011-07-21

**Authors:** Favio Bortoletto, Carlotta Bonoli, Paolo Panizzolo, Catalin D. Ciubotaru, Fabio Mammano

**Affiliations:** 1 Istituto Nazionale di Astrofisica, Osservatorio Astronomico di Padova, Padova, Italy; 2 Fondazione per la Ricerca Biomedica Avanzata, Istituto Veneto di Medicina Molecolare, Padova, Italy; 3 Università di Padova, Dipartimento di Fisica, Padova, Italy; 4 Consiglio Nazionale delle Ricerche, Istituto di Neuroscienze, Padova, Italy; University of Manchester, United Kingdom

## Abstract

Graded Index (GRIN) rod microlenses are increasingly employed in the assembly of optical probes for microendoscopy applications. Confocal, two–photon and optical coherence tomography (OCT) based on GRIN optical probes permit in–vivo imaging with penetration depths into tissue up to the centimeter range. However, insertion of the probe can be complicated by the need of several alignment and focusing mechanisms along the optical path. Furthermore, resolution values are generally not limited by diffraction, but rather by optical aberrations within the endoscope probe and feeding optics. Here we describe a multiphoton confocal fluorescence imaging system equipped with a compact objective that incorporates a GRIN probe and requires no adjustment mechanisms. We minimized the effects of aberrations with optical compensation provided by a low–order electrostatic membrane mirror (EMM) inserted in the optical path of the confocal architecture, resulting in greatly enhanced image quality.

## Introduction

Optical microscopy for *in vivo* analysis deep within tissues requires the application of a series of well known observational techniques (confocal, two–photon fluorescence or OCT) supported by small and non–invasive optical probes. GRIN rod lenses guide light using internal variations in the refractive index rather than the curved refractive surfaces employed by conventional lenses [1]. They can be assembled in a sequence of typically 1–3 elements to form a microendoscope probe that acts essentially as an optical relay (http://www.grintech.de/gradient-index-optics.html). GRIN probes for microendoscopy are typically combined with more classic optical elements such lenses [2]. GRIN rod microlenses, can be modeled as conventional optical elements, aiding the design of miniaturized objectives. Approximate ranges of typical values for microendoscopy probes based on GRIN microlenses are: 0.5–3 cm for physical lengths; 150–800 µm for optical working distances; 0.4–0.75 for numerical apertures (NAs) and 100–1000 µm for fields of view [3]. Even probes with sizes down to 125 µm have been mounted inside hypodermic needles (310 µm outer diameter) to create flexible and non–invasive optical probes [4]. A probe with an external diameter of 360 µm and carefully engineered fiber structure was able to provide optical manipulation and analysis of microscale specimens [5].

Applications for in–vivo two–photon fluorescence microscopy using scanned GRIN probes have been used for basic research [2], as well as for histological guidance during resection of brain tumors [6]. Owing to their small size, GRIN microendoscopy probes can be incorporated into miniaturized two–photon microscopes [7], [8], [9], [10], [11]. Also OCT applications based on GRIN probes coupled to micro–mechanical (MEM) scanning mirrors have been presented [12].

In our prior work we described a compact infinity–corrected GRIN objective, with 0.5 numerical aperture (NA) in water, suitable for microendoscopy (0.5 mm diameter), which we assembled from commercially available components [13]. In the present study we used a low–order adaptive optics (AO) system to minimize static distortions and intrinsic low–order aberrations that limit the performance of GRIN based microendoscopy obiectives coupled to multiphoton microscopes.

## Results

### System construction

**Figure 1:**
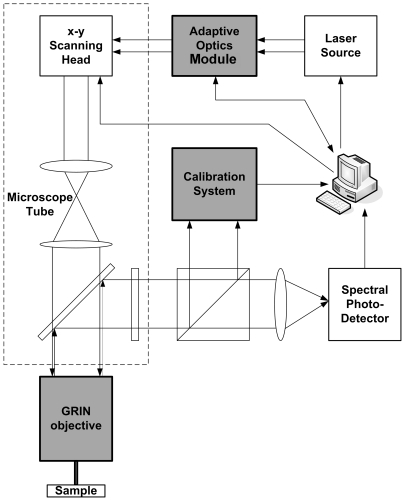
Scheme of the apparatus. A standard two–photon fluorescence microscope was modified by insertion of an AO module, a PSF calibration system and a GRIN fiber objective (grey boxes).

Figure 1 shows a diagram of our imaging apparatus, which was retro–fitted to a commercial two–photon laser scanning confocal microscope. The devices inserted on the microscope optical path, shown as grey boxes in Figure 1, comprised:

1. an AO module based on an EMM
2. a GRIN fiber objective mounted on the microscope standard objective receptacle
3. a calibration system based on an imaging camera illuminated by an optical relay reproducing a magnified view of the field covered by the GRIN objective

**Figure 2:**
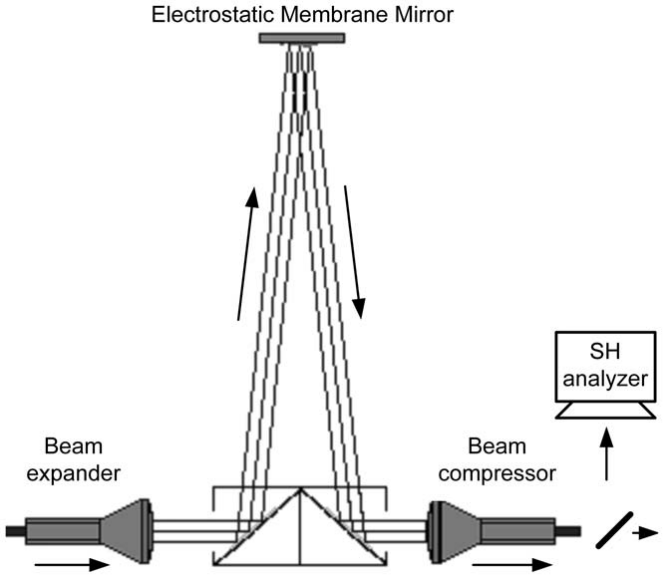
Adaptive optics module. Detailed diagram of the AO module interposed on the input laser beam before the microscope scanning head.

An important constraint in this AO application was to maintain the same adaptive mirror correction action, in terms of impulse response function, over the whole view field explored by the microscope scanning head, avoiding light vignetting. In principle, one could recreate a suitable field invariant pupil after the confocal scanning head using custom designed optics. Unfortunately this proved impossible in our configuration, due to constraints imposed by the underlying commercial architecture. The simplest solution, and the one we adopted, was to intercept the laser beam before it entered the scanning head (Figure 2), at the expense of some criticality in terms of mirror positioning and adjustments due to the resulting long optical leverage. To ease the fitting procedure, the AO module was placed on a sturdy platform with micrometric movements in 3 orthogonal directions. The Gaussian laser beam, with full width at half maximum (FWHM) of about 2 mm, average power of 350 mW and central wavelength of 830 nm, was directed onto the AO module and thereafter reinserted on its ordinary light path. The laser beam cross–section was adapted to the working surface of the EMM (10 mm diameter) by a beam expander (3×) followed by a complementary beam compressor after reflection off the mirror (Figure 2).

**Figure 3:**
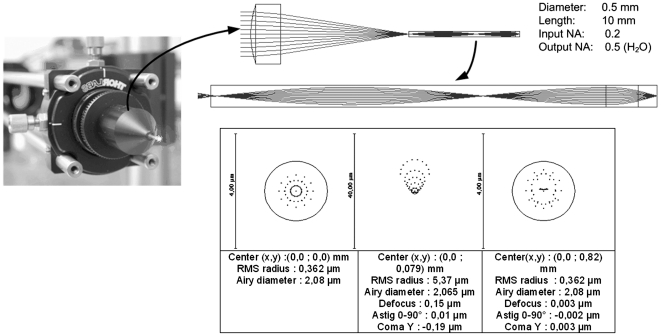
GRIN objective. Top: the objective was assembles using a commercial aspheric lens coupled to the GRIN fiber and embodied in an anodized aluminum mount with a standard microscope collar. Bottom: Zemax spot diagrams for the Deformable Mirror plus GRIN objective combination in water; left box, the nominal on–axis Airy disk is about 2 µm; center box: beam distortion at 80 µm from center; right box: simulated correction performed by the EMM.

A view of the mounted objective with its main specifications is presented in Figure 3. Note that a Zemax model predicts an Airy radius of 2 µm for the PSF of the objective when immersed in water (Figure 3). Therefore, if the Zemax representation of the GRIN element were sufficiently accurate, this would be the order of the expected resolution of the system in the absence of other distortions and aberrations. As a consequence of back–lash between fiber and the capillary tube holder (wall to wall free space of about 0.05 mm) and positioning errors of the capillary on the objective aluminum body, an unavoidable combined optical axis misalignment was present between GRIN fiber and illuminating lens. This introduced a certain amount of optical distortion, as derived from a tolerance analysis made on the Zemax model limited to low order terms, namely: defocusing and spherical aberration, astigmatisms and comas. Theory for deformable, continuous membranes fixed at the border (our electrostatic mirror) [14] shows that these aberrations can be corrected by the AO module, possibly with the only exception of spherical aberration where the mirror can suffer of relatively small modal gain. Likewise, the distance between fiber and illuminating aspheric lens, mounted in the same fiber support (Figure 3), was pre–adjusted during the objective assembly. This lack of mechanisms for further focal optimization was dictated by our aim to construct a compact objective in which most of the fiber length (8 mm) could be inserted in live specimens. Also these distortions should be ultimately taken care of by the AO module. Thus in our view, and in our experience with this peculiar application of GRIN objectives to multiphoton microscopy, AO serves the dual purpose of (i) facilitating system assembly, by correcting static optical distortions due to residual alignment criticalities, and (ii) correcting low order aberrations that are intrinsic in the optical components.

The calibration system was assembled using commercial relay optics and inserted on the fluorescence beam returned by the GRIN objective before the photomultipliers that formed the so called *direct detection system* (DDS) of the confocal microscope. The calibration system imaged the central portion of the optical field viewed by the objective onto a CMOS camera with a magnification factor of 43.5 (determined using a calibration target). An important aspect of the AO application described here was the pre–calibration of the EMM flexural modes (influence matrix), which was required by the optimization algorithm (described in the next section). This calibration needed to be performed once and for all and provided the full description of the EMM. To perform the calibration we used a Shack–Hartmann camera (SHC) to reconstruct the mirror influence matrix. With reference to Figure 2, the SHC was directly interposed on the collimated beam emerging from the output beam compressor and thus it worked with the same optical configuration used at run time. It should be noted that recent commercial mirrors are directly provided with their peculiar influence matrix.

### System calibration and performance

In a traditional AO application, a classical example is the astronomical case [15], the impinging wavefront phase is retrieved in real–time via a wavefront analyzer (pure phase or modal form) profiting from an in–field reference source (usually a star). Closed–loop control of a deformable optical element is then possible. In contrast, direct wavefront error sensing in microscopy applications is hampered by lack of a suitable reference, as well as lack of a true sub–divisible pupil. Consequently modal wavefront error estimation can be obtained only indirectly, by deliberately introducing known amounts of modal distortion [16]. Alternatively, the whole optical system must be previously optimized on a calibration configuration to be later applied during observation. The latter is the approach used here to minimize the effects of static optical distortions. The procedure we used was based on two off–line steps:

1. *Calibration*: making use of the SHC, retrieve the EMM *solicitation matrix* and the low order Eigenvectors (*flexure modes*) with corresponding electrode voltages patterns.
2. *Optimization*: iteratively determine the optimal EMM set–up suitable for different observational situations using a gradient descend algorithm acting on weighted combinations of the retrieved characteristic voltages patterns.

The first operation is detailed in [13] and [17]; a simplified discussion, which is required to introduce our method, is reported hereafter.

**Figure 4:**
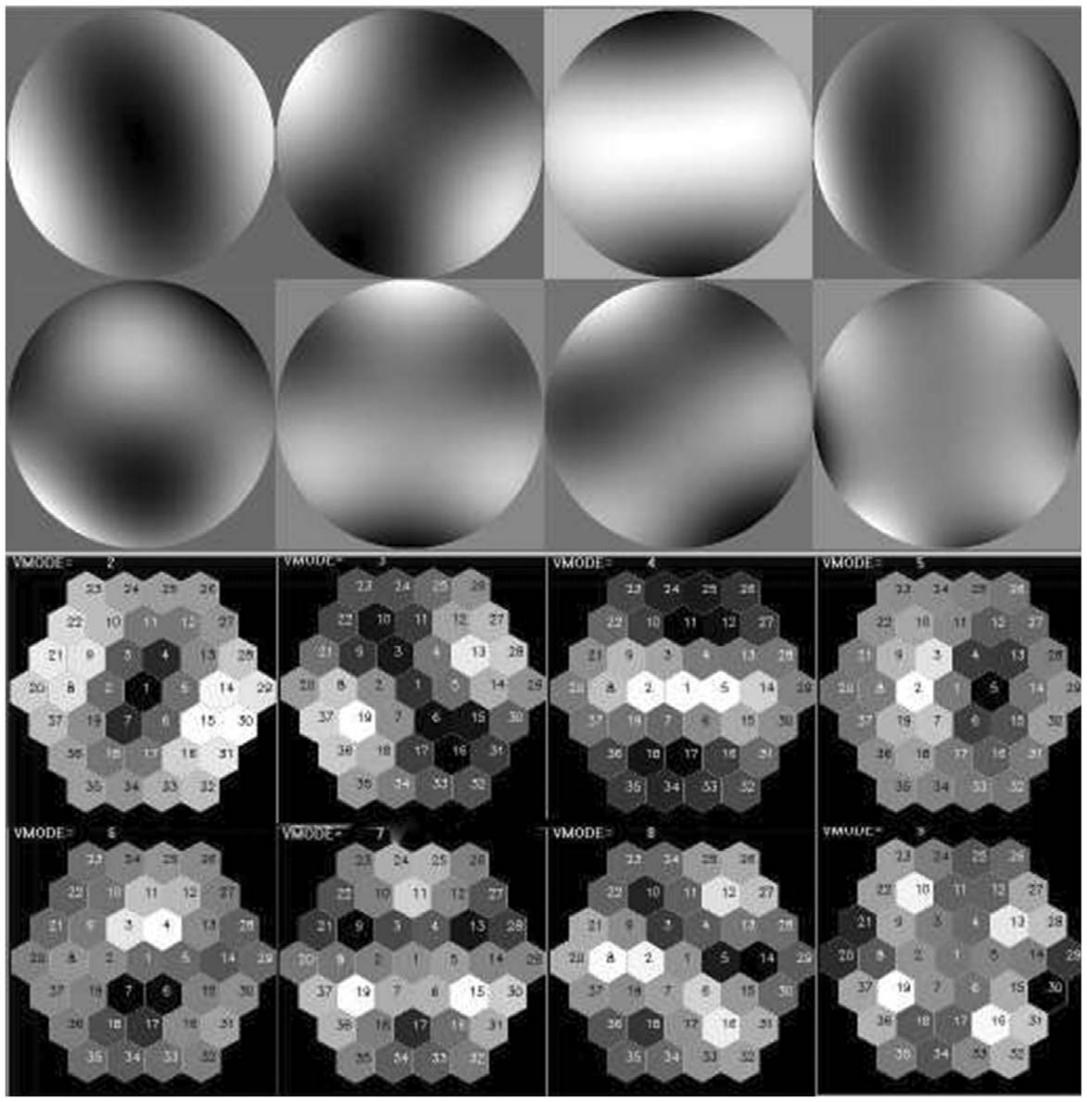
Flexure modes and corresponding voltage patterns. First eight flexure modes (top) and corresponding voltage patterns (bottom) used for PSF optimization. Modes were generated by sequentially pulsing each electrode at minimum voltage and recording the corresponding wavefront phase with the Shack–Hartmann camera.

After construction, the *solicitation matrix* 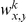 was decomposed by singular value decomposition (SVD) in:
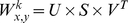
(1)where:

*U* is an orthogonal matrix 37×(256×256)
*S* is a diagonal matrix 37×37
*V^T^* is an orthogonal matrix 37×37

and with index *k* spanning the 37 EMM electrodes, whereas *x* and *y* are the discrete coordinates (256×256 pixels) of the phase images produced by the SHC.


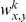
 is usually inverted to create an *influence matrix*:
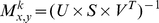
(2)suitable for a modal correction loop. Instead, we focused on the set of matrices *U*, *S* and *V^T^*. It is well known that the matrix *U* contains the EMM membrane *flexure modes* in increasing order, the matrix *V* the corresponding solicitation patterns while the *S* diagonal represents the transfer ‘gain’ for each flexural mode [14]. In the present case, the resulting modes and corresponding voltage patterns are presented in Figure 4. The flexure modes obtained in this way are orthogonal and their voltage–pattern counterpart can be directly used to optimize the system. This procedure does not require the use of Zernike polynomials [18], [19], with the resulting benefit of a more compact and efficient minimization algorithm.

The second step, the iterative optimization process of the point spread function (PSF), required the immersion of the GRIN objective in a fluorescent solution to retrieve an image of the PSF on the calibration system camera. The key idea is to minimize PSF image size in a loop, whereby low order flexure modes weight factors are perturbed and the resulting PSF is estimated. Iterative minimization was based on a merit factor extracted from each image after a linear fit of a two–dimensional Gaussian to the fluorescence spot profile recorded by the calibration camera:
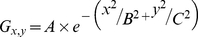
(3)The merit factor ε, a decreasing quantity, was then computed by combining the Gaussian width in the *x* and *y* directions, namely the parameters B and C in Equation 3, normalized by the peak value, P*_val_*, of the fitted PSF image intensity:
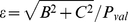
(4)


The optimization metric described by Eq. 4 was preferred because it involves both the intensity and shape of the sampled PSF. The Strehl ratio, the most obvious choice, was considered unsuitable because strictly dependent on PSF intensities, which were relatively unstable due to noise in the detected signal during long optimization trials. Compared to simple ‘spot size’ measurements, the merit factor ε proved to be less susceptible to noise and provided the most stable and fast convergence. In order to minimize the camera noise pattern error, ε was computed on running groups of ten consecutive PSF images. ε was than used by a C code implementation of the classical downhill simplex minimization method [20] working on 8+1 variables, where the first 8 were weight factors for the flexural modes.

**Figure 5:**
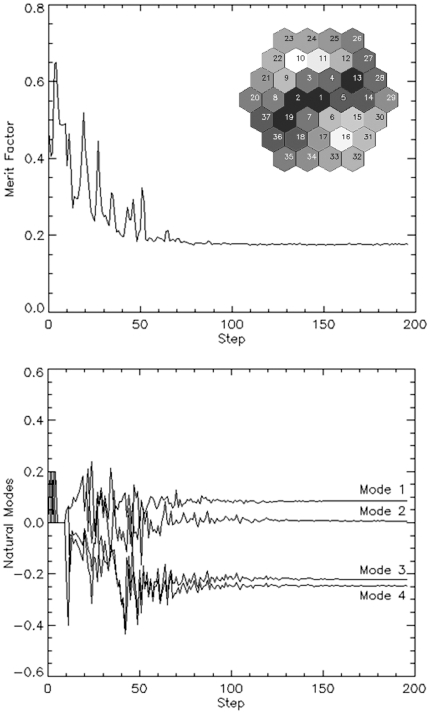
Merit factor and final mirror pattern. Behavior of merit factor ε (top) and of first four eigenmodes weight factors (bottom) versus minimization step. The final pattern commanded to the EMM after optimization is shown as inset in the top panel.

**Figure 6:**
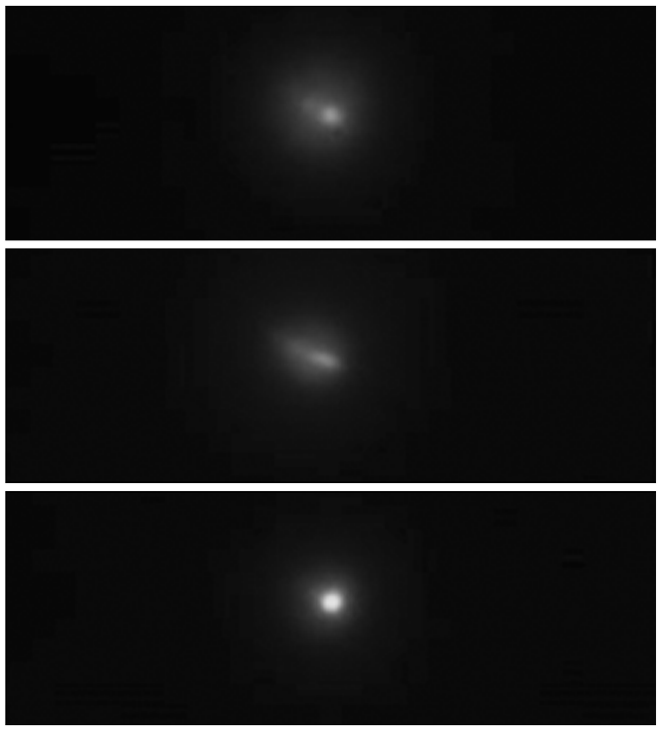
Images of the point spread functions captured at crucial steps during the calibration procedure. Top, AO module not inserted in the optical path; middle: module inserted with EMM at rest position; bottom: typical result after AO optimization.

Minimizations were tested both using the first eight EMM flexure modes, as well as using just the first four, with similar results; results of a typical PSF minimization trial with four modes are shown in Figure 5. Finally, we tested the stability of the PSF optimization system by reiterating the whole procedure 10 times. Table 1 lists mean ± standard deviation (S.D.) results obtained starting from the same EMM configuration, i.e. with electrodes all fixed at a value of 2^12^ √2 = 2895 corresponding to about 226 Volts. Coefficients stabilized typically after 50–70 iterations in a total of approximately 400 s using eight flexure modes. Typical PSF images obtained in the course of one of the 10 trials are presented in Figure 6. The middle panel in Figure 6 highlights the typical degradation undergone by the PSF following insertion of the EMM, mainly due to the natural astigmatism of the membrane at rest. It should also be noted that the PSF shape is never exactly Gaussian. Perhaps it could be better simulated by the sum of two coaxial Gaussian functions with different parameters, i.e. a low and wide background plus a narrow and taller spike. This is visible in Table 2, where the FWHM is estimated both on the PSF shape and on the Gaussian fit. Ellipticity in Table 2 is defined as the ratio between minor and major FWHM of the fitted Gaussian profiles; it demonstrates the action of AO in profile regularization.

**Table 1:** System stability: Average values and dispersions obtained after ten independent optimizations.

	Merit Factor ε	Mode 1	Mode 2	Mode 3	Mode 4
Average Value	1,898	0,108	−0,047	−0,179	−0,200
RMS	0,011	0,065	0,022	0,069	0,041

**Table 2:** Average results obtained from a series of ten on axis optimizations from fluorescence solution images collected at the calibration arm.

	No AO	AO at rest	AO
FWHM µm	2.39	3.71	1.98
FWHM Gaussian fit µm	3.46	5.09	2.88
Ellipticity	0.91	1.14	0,98

**Figure 7:**
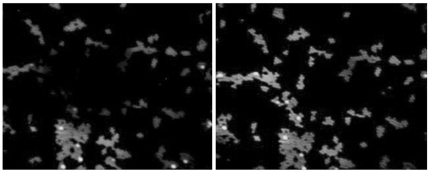
Images of 1 µm fluorescent microspheres at low magnification. Whole view field before (left) and after (right) AO correction with parameters retrieved during optimization. The field spans about 80×80 µm.

**Figure 8:**
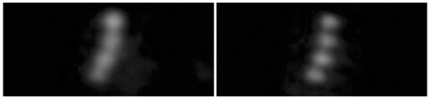
Images of 1 µm fluorescent microspheres at higher magnification. A chain of four 1 µm fluorescent spheres imaged before (left) and after (right) AO application.

As a final test of our imaging apparatus, we imaged fluorescent micro–beads (1.0 µm diameter, peak emission around 515 nm) excited by the two–photon laser tuned at 830 nm with and without AO assist. As shown in Figure 7, the PSF was clearly sharpened and regularized independent of the position in the scanned field (about 80×80 µm). Figure 8 shows a different enlarged field at higher magnification. Notice that PSF sharpening and regularization allowed the detection of the minute spaces between adjacent spheres. The degree of sharpening was compatible with the results reported in Table 2. Tests performed with 0.5 µm beads show that this is close to the resolution limit of the system (Figure S1).

## Discussion

Thanks to microendoscopy, the penetration depth of laser–scanning microscopy into tissue can be increased up to the centimeter range [3]. When a microendoscopy probe is coupled to a (multiphoton) laser scanning microscope, the laser focal spot is scanned just above the surface of the probe that lies outside tissue and the probe projects a the scanning pattern to a focal plane inside the tissue. Optical aberrations that are intrinsic to the endoscope probes, and also due to imperfect coupling to the feeding optics, have limited the resolution to 0.9–1.2 µm and 10–12 µm in the lateral and axial directions, respectively [8], [9], [21]. Low order adaptive optics (AO) applications have been previously described within standard microscopy environment [16], [19], [22], as well as in simultaneous optical sectioning and manipulation [23].

In the current study, we coupled a compact GRIN fiber objective, described in our prior work [13], to a two–photon microscope retrofitted with a commercially available AO system. Our results indicate that AO can be exploited to approach diffraction–limited performance with GRIN microendoscopes. The application required the definition and set–up of:

- a calibration procedure and optical architecture to insert the AO components in the microscope optical path
- a computer algorithm to optimize the performance of the system

Tests with calibrated samples highlighted:

- compatibility with the results obtained during system optimization phase
- coverage of the full field of view with a corrected and uniform PSF.

Two critical factors limited the performance of our system, both due to the necessity of interfacing the AO module with a commercial multiphoton architecture. Firstly, the mismatch between the diameter of the EMM and that of the incoming laser beam forced us to introduce a beam expander and a corresponding beam compressor in the light path preceding the scanning head of the microscope. These extra optical components reduced the power of the laser beam reaching the sample and significantly complicated the alignment of the system. These problems can be solved using MEMS based mirrors with smaller and more numerous active elements, which would not require modification of the laser beam. The second critical point was the poor photon–capture capability of the commercial two–photon microscope, which was primarily due to the native remote positioning of the photodetectors, far away from the back focal plane of the objective. Also this limitation could be easily circumvented by a suitable redesign of the optical paths, as well as by adopting state–of–the art photosensors. Despite these shortcomings, the tests we performed with fluorescent microspheres clearly indicate that AO can substantially improve image quality by minimizing the effects of intrinsic and extrinsic low order aberrations in fluorescence multiphoton microendoscopy.

## Materials and Methods

FluoSpheres® carboxylate–modified microspheres, 1.0 µm, yellow–green fluorescent, with peak emission around 515 nm (Cat. N. F–8823, Invitrogen) were imaged by the system described above a retro–fitted to Biorad Radiance 2100 confocal microscope mounted on a Nikon Eclipse 600 upright fluorescence microscope and fed by a mode–locked Ti:Sapphire laser (Tsunami, Spectra–Physics/Newport Corporation, Irvine, CA, USA).

The naked GRIN fiber was purchased from GRINTECH Gmbh. It was based on a ¼ pitch device with a 0.5 in–water NA coupled to a ¾ pitch relay lens with a 0.2 NA entrance. The overall probe (0.5 mm diameter) was inserted and epoxy glued in an steel capillary tube (0.7 mm outer diameter and 10 mm length) for light shielding and mechanical protection.

The AO module was based on an EMM with 37 electrodes controlled by high voltage amplifiers and interfaced to a PC using a 12–bit USB controller (Flexible Optical B.V., Rijswijk ZH, The Netherlands).

## Supporting Information

Figure S1 **Images of 0.5 µm fluorescent microspheres.** Left: sample of 0.5 µm fluorescent spheres (Cat. N. F–8813, Invitrogen) imaged with the GRIN objective after AO application; right, a different field of the same sample imaged with a high NA commercial water immersion objective (UApoN340 40×1.15 NA, Olympus) without AO intervention.
(TIF)
