# Supplementary figures and images for: Multiphoton Fluorescence Microscopy with GRIN Objective Aberration Correction by Low Order Adaptive Optics
Source: PLoS One. 2011 Jul 21;6(7):e22321. doi: 10.1371/journal.pone.0022321 (PMC3141032; doi:10.1371/journal.pone.0022321)

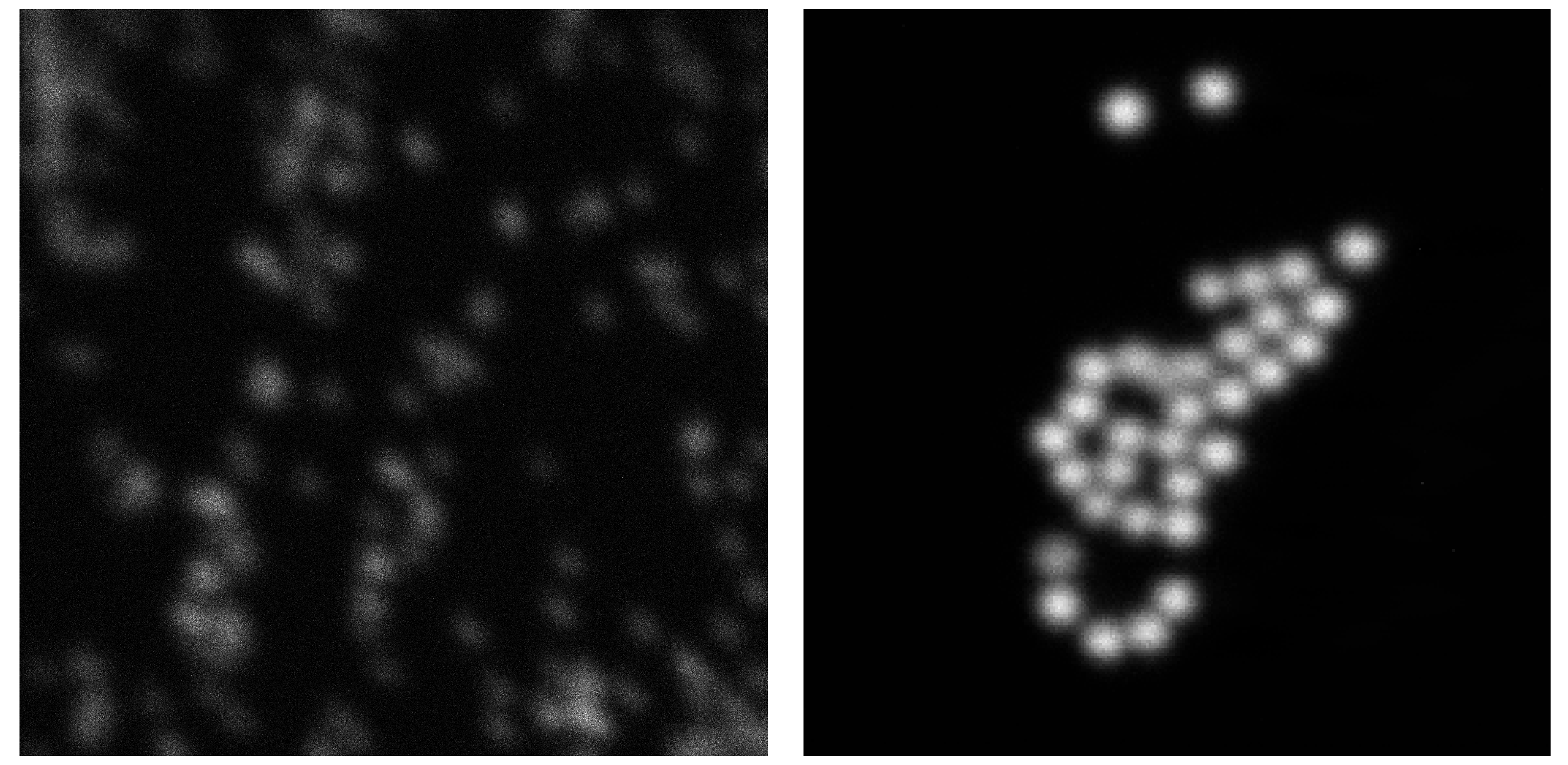

Supplement: Figure S1 — Images of 0.5 µm fluorescent microspheres. Left: sample of 0.5 µm fluorescent spheres (Cat. N. F–8813, Invitrogen) imaged with the GRIN objective after AO application; right, a different field of the same sample imaged with a high NA commercial water immersion objective (UApoN340 40×1.15 NA, Olympus) without AO intervention. (TIF) [file pone.0022321.s001.tif]
